# Arterial supply and morphological characteristics of sympathetic neurons in the human superior cervical ganglion

**DOI:** 10.3389/fnana.2024.1372180

**Published:** 2024-03-06

**Authors:** Jelena Boljanović, Milan Milisavljević, Milan Latas, Laslo Puškaš, Nikola Bogosavljević, Marko Vujačić, Dejan Aleksandrić, Dejan Ćetković, Nemanja Branković, Aleksandra Dožić, Mila Ćetković

**Affiliations:** ^1^Laboratory for Vascular Morphology, Institute of Anatomy, Faculty of Medicine, University of Belgrade, Belgrade, Serbia; ^2^Academy of Medical Sciences, Serbian Medical Association, Belgrade, Serbia; ^3^Clinic for Psychiatry, University Clinical Center of Serbia, Faculty of Medicine, University of Belgrade, Belgrade, Serbia; ^4^Institute for Orthopedic Surgery “Banjica”, Faculty of Medicine, University of Belgrade, Belgrade, Serbia; ^5^Institute of Anatomy, Faculty of Dental Medicine, University of Belgrade, Belgrade, Serbia; ^6^Pacemaker Center, University Clinical Center of Serbia, Faculty of Medicine, University of Belgrade, Belgrade, Serbia; ^7^Institute of Histology and Embryology, Faculty of Medicine, University of Belgrade, Belgrade, Serbia

**Keywords:** micromorphological analysis, ganglionic neurons, mast cells, microvessels, superior cervical sympathetic ganglion

## Abstract

The aim of this study was the micromorphological analysis of the distribution of microvessels, mast cells and ganglionic neurons in two parts, proximal and distal of the human superior cervical sympathetic ganglions (SCSGs). Statistical analyses were applied to detect the possible metric regional differences in their densities. Five injected human SCSGs with colored India ink and gelatin were microdissected and examined. Second group of five human SCSGs was prepared and serially sliced for CD34 and mast cell tryptase immunostaining. The microscopic fields of two parts of the SCSGs were analyzed for the following quantifications: microvessel density (MVD), mast cell density (MCD), and ganglionic cell count and measurements. The mean number of CD34-positive microvessels in microscopic fields, the MVD, had a value of 83 for the upper parts, and 82.7 for the lower parts of SCSGs. The mean number of tryptase-positive mast cells in microscopic fields, the MCD, was 4.5 in the proximal parts, and 4.7 in the distal parts of SCSGs. The mean number of ganglionic neurons in microscopic fields was 19.5 in the proximal parts, and 19.8 in the distal parts of SCSGs. The density of CD34-positive microvessels, the density of tryptase-positive mast cells, and the density, mean diameters and mean areas of ganglionic neurons were not significantly different in two observed parts, upper and lower of the SCSGs. In conclusion, the distributions of microvessels, mast cells, and neurons in two parts of the SCSGs were uniform with no specific micromorphological variations, there is a homogenous vascular and cellular pattern within the SCSGs.

## Highlights

- The reason for this investigation was the question of whether within the human superior cervical sympathetic ganglion (SCSG) there is an uneven layout of blood vessel network and of mast cells, which could possibly influence the nerve cells and axons in a specific portion of the ganglion.
- By means of immunohistochemical staining, we depicted the intraganglionic microvessels as well as mast cells within the SCSG.
- To our knowledge, there are no reports on the density of microvessels in the human SCSG, on the number of mast cells within the ganglion, or on the number and measurements of ganglionic cells.
- The intraganglionic network of blood vessels and clusters of ganglionic neurons are uniformly arranged within the SCSG. The intraganglionic mast cells are numerous and uniformly present, possibly with the potential of modulating the function of sympathetic ganglionic cells.

## Introduction

The ganglia of the peripheral nervous system (PNS): sympathetic, parasympathetic and sensory, are composed of morphologically and functionally different neurons. The ganglia of the PNS are collections of nerve cell bodies enveloped in a sheath of satellite glial cells. Satellite glial cells (SGCs) are specialized cells that form a morphological and functional perineuronal enclosure around neurons in the PNS ganglia (sympathetic, parasympathetic and sensory) and maintain normal neuronal homeostasis (Milosavljević et al., 2021; Zdora et al., 2022). Dural and superficial cerebral blood vessels receive innervation from the ganglionic cells within the ganglia of the PNS. Sympathetic perivascular nerves originate from the SCSG, release neuropeptide Y (NPY), adenosine triphosphate (ATP), and norepinephrine, and produce vasoconsriction. Parasympathetic nerves come from the pterygopalatine and otic ganglions and contain vasoactive intestinal peptide (VIP), nitric oxide (NO) and acetylcholine (ACh), and act as strong vasodilators. Sensory nerves are peripheral processes of pseudounipolar trigeminal ganglionic cells with substance P (SP) and calcitonin gene-related peptide (CGRP) (Hamel, 2006; Agarwal et al., 2021).

The superior cervical sympathetic ganglion (SCSG) is the first and the largest of the three cervical sympathetic ganglia. It lies lateral to the pharyngeal wall on the longus capitis muscle, over the transverse process of second cervical vertebra (C2), posterior to the contents of carotid sheath: the internal carotid artery, and the vagus nerve and internal jugular vein more laterally (Fazliogullari et al., 2016). The ganglionic sympathetic neurons are multipolar with postganglionic unmyelinated axons. Micromorphological specificity of the SCSG is distribution of ganglionic sympathetic neurons in two zones, upper (proximal) and lower (distal) related to the origin of axons of the internal carotid nerve (upper part) or the external carotid nerve (lower part). The internal carotid nerve exits from the upper pole of SCSG and follows the internal carotid artery for the innervation of upper head and intracranial vasculature. The external carotid nerve and laryngopharyngeal branches of the SCSG provide sympathetic postganglionic innervation to the lower head and neck organs, and the superior cervical cardiac branches have positive effects to the cardiovascular functions (Headley et al., 2005; Standring, 2021).

The potential participation of the sympathetic system in the development of headache is still not explained, but the autonomic reactions in specific organs (e.g., pupils, sweat glands, heart, etc.) which follow the pain could be the result of sympathetic activity. Axons of the internal carotid nerve of the SCSG could be compressed during dilatation of the internal carotid artery inside the carotid canal, generating signs of sympathetic lesion (Hoffmann et al., 2019). Norepinephrine from the sympathetic nerves by coupling with sensory neurons participates in stimulation or sensitization of nociceptive nerves, this is known as “sympathetically maintained pain” (Jänig, 2003).

The ascending pharyngeal artery is the first and thinnest branch of the external carotid artery. It ascends between the pharynx and internal carotid artery sending branches for the supply of prevertebral muscles, the pharyngeal wall, the tympanic cavity, and the sympathetic trunk (Tubbs et al., 2002). Intraganglionic microvessels, forming a network of vessels, curve over the layer of SGCs, which completely cover the surface of ganglionic neurons with a gap of 20 nm in between. This unique “neuron-glial unit” with adjacent covering of mostly capillaries, represents “the blood-nervous tissue barrier of the peripheral nervous system” (homolog of the central nervous system blood–brain barrier) (Ćetković et al., 2020; Hanani and Spray, 2020; Milosavljević et al., 2021).

The optimal distribution of intraganglionic blood vessels is of crucial importance for the functional stability of this specific neural network of the SCSG and SGCs. Irregularities in the branching pattern of intraganglionic small vessels may lead to changes in SGCs. The SGCs activation, because of the communication with the neurons of SCSG and the synapses over the multipolar perikarya, leads to resulting neuronal dysfunction. This may generate lack of control of the synaptic transmission by the SGCs, and hyperexcitability of sympathetic cell bodies, causing pain as a result of exacerbated neuronal activity (Hanani and Spray, 2020). We do not know the exact intraganglionic distribution of microvessels of the SCSG, compared to existing descriptions of the supply of the trigeminal and geniculate ganglia (Smoliar et al., 1998; Dožić et al., 2014; Ćetković et al., 2020; Mirčić et al., 2021).

Different studies suggested an important involvement of meningeal mast cells as a major source in triggering migraine pain. Thus, mast cells are normally present in the sensory and sympathetic ganglia, and also in periganglionic fibrous tissues, in close relations to both nerve fibers and vessels. Possible damage to ganglionic neurons and the release of ATP from different cells, or glutamate from SGCs in the TG, may lead to activation and degranulation of resident mast cells with the release of substances such as the most important serotonin, histamine, and pro-inflammatory cytokines, which seem to play a relevant role in the activation of meningeal and vascular nerve fibers, and a migraine attack or development of pathologic pain (Koroleva et al., 2019; Koyuncu Irmak et al., 2019; Hanani and Spray, 2020; Cho et al., 2023).

One reason for this study is the neurological and surgical significance in understanding the ganglionic neurovascular anatomy and the current lack of relevant anatomic data (Siegenthaler et al., 2013). The SCSG is in charge with the sympathetic innervation of structures of the head and neck (blood vessels, dura mater, pineal gland, organs in the orbit, salivary and thyroid glands, carotid body, heart) (Sapio et al., 2020; Standring, 2021). Injection of tracers into the different target organs of the head and neck and analysis of retrogradely labeled neurons of the SCSG showed a relatively precise topographical distribution of neurons related to the rostrocaudal axis. Rostral part of the SCSG sent axons to the upper part of the head (pineal gland, forehead, eyes). The caudal part of the SCSG neurons projected to the mouth and neck structures (Flett and Bell, 1991). Another study showed the relation between the rostro-caudal position, size and immunoreactivity for neuropeptide Y (NPY) in the rat SCSG neurons. Results indicated that NPY-positive neurons are smaller than NPY-negative neurons at more distal position in the SCSG (Headley et al., 2005).

Our goal was to analyze microvessel density (MVD), mast cell density (MCD), as well as the micromorphometric characteristics of neurons of two parts, proximal and distal, of the human SCSG, using immunohistochemical staining and image analysis software. The correlation between the examined parameters has been established.

## Materials and methods

### Microanatomical examination

Five adult human superior cervical sympathetic ganglions (SCSGs) were used for the micromorphological analysis of their peri and intraganglionic arterial branching pattern. After perfusion of the carotid arterial systems with isotonic saline solution, we injected into the common carotid artery a mixture of 10% water solution of colored India ink and melted gelatin. The cervical arteries were microdissected and examined under the stereo microscope. Sections of one injected SCSGs were cleared and made transparent by a modified Spalteholz technique and analyzed under transmitted light.

### Histological and immunohistochemical examination

For the immunohistochemical study we used five human SCSGs of adult persons taken after the disarticulation of the head and reflection of the pharyngeal wall from the vertebral column. After removal each ganglion was immediately immersed in isotonic saline solution, fixed in 4% buffered formaldehyde, dehydrated, embedded in paraffin and sectioned serially in 4 μm thick slides. Every tenth slide was stained with hematoxilin and eosin (H&E) stain, the next two with the Masson trichrome method and silver staining method, and the next two slices were prepared for immunohistochemical procedure. We prepared 10 sets of slices from every ganglion. All slices for the immunohistochemistry were first deparaffinized and then the sections were treated for antigen retrieval prior to staining. The endogenous peroxidases were blocked by incubating the samples with 3% hydrogen peroxide solution. The slices underwent the immunostaining by the incubation with the following mouse monoclonal primary antibodies; against CD34 (DAKO A/S, Denmark M 7165, 1:25), and anti-mast cell tryptase (DAKO A/S, Denmark M 7052, 1:100). The sections with the bound antibodies, which had to be visualized, were stained with a Mouse/Rabbit PolyDetector DAB HRP Brown (Bio SB) detection system. The sections were counterstained with Mayer’s hematoxylin, dehydrated, and covered with cover slips. The intensity of staining was evaluated semiquantitatively by 2 independent investigators. The intensity of all the slices was classified as strongly positive (+++). Negative controls, for assessing nonspecific staining, were performed by incubating slices with non-immune serum, in order to determine the specificity of immunostaining. The slices were analyzed with a light microscope (Leica DMLS), and photomicrographed by a digital photocamera (Leica DFC295). Measurements were performed using image analysis software (Leica Interactive Measurements). The study protocol was approved by the Ethics Committee of the Faculty of Medicine (No. 1322/VII-23, Date 07.07.2022).

### Morphometric study

Microvessel density (MVD) was defined as the mean number of microvessels visible in microscopic fields of analyzed tissue. The microvessels, mainly capillaries and precapillaries, with diameters from 5.5 μm to 8.5 μm, were identified by immunostained parts of their vascular walls or variously transected independent vessels. The number of the microvessels was counted in ten microscopic fields of SCSGs parts, proximal and distal, at x400 magnification (objective lens x40 and ocular lens x10). The arithmetic mean of the 10 fields of each of 5 ganglions, measuring 341.7 μm x 250.0 μm in size each, with a corresponding area of 85,425 μm^2^ (0.085 mm^2^) per field, was calculated for microvessel number. We also counted the number of immunostained mast cells for calculating the mast cell density (MCD) in two established parts and microscopic fields, proximal and distal, of the SCSGs. A similar procedure was applied separately for counting multipolar ganglionic neurons in each field of the mentioned slices. The longer and shorter diameters were measured and the mean diameter calculated, the circumference and cross-sectional area (μm^2^) measured and calculated. In our measurements we included only cross-sectional areas of neurons sectioned through the visible cell nuclei. The size of perikarya was measured in 195 ganglionic neurons of proximal and 198 ganglionic neurons of distal parts of five SCSGs.

### Statistical analysis

Quantitative experimental data were analyzed using the IBM SPSS Statistics 25.0 statistical software package (SPSS, Inc., Chicago, IL, United States). The statistical analyses comprised mean values and standard deviations (M ± SD), *T*-tests for independent samples, and one-way analysis of variance (ANOVA). The probability level of *p* < 0.05 was considered an appropriate indication of statistically significant difference.

## Results

**Figure 1:**
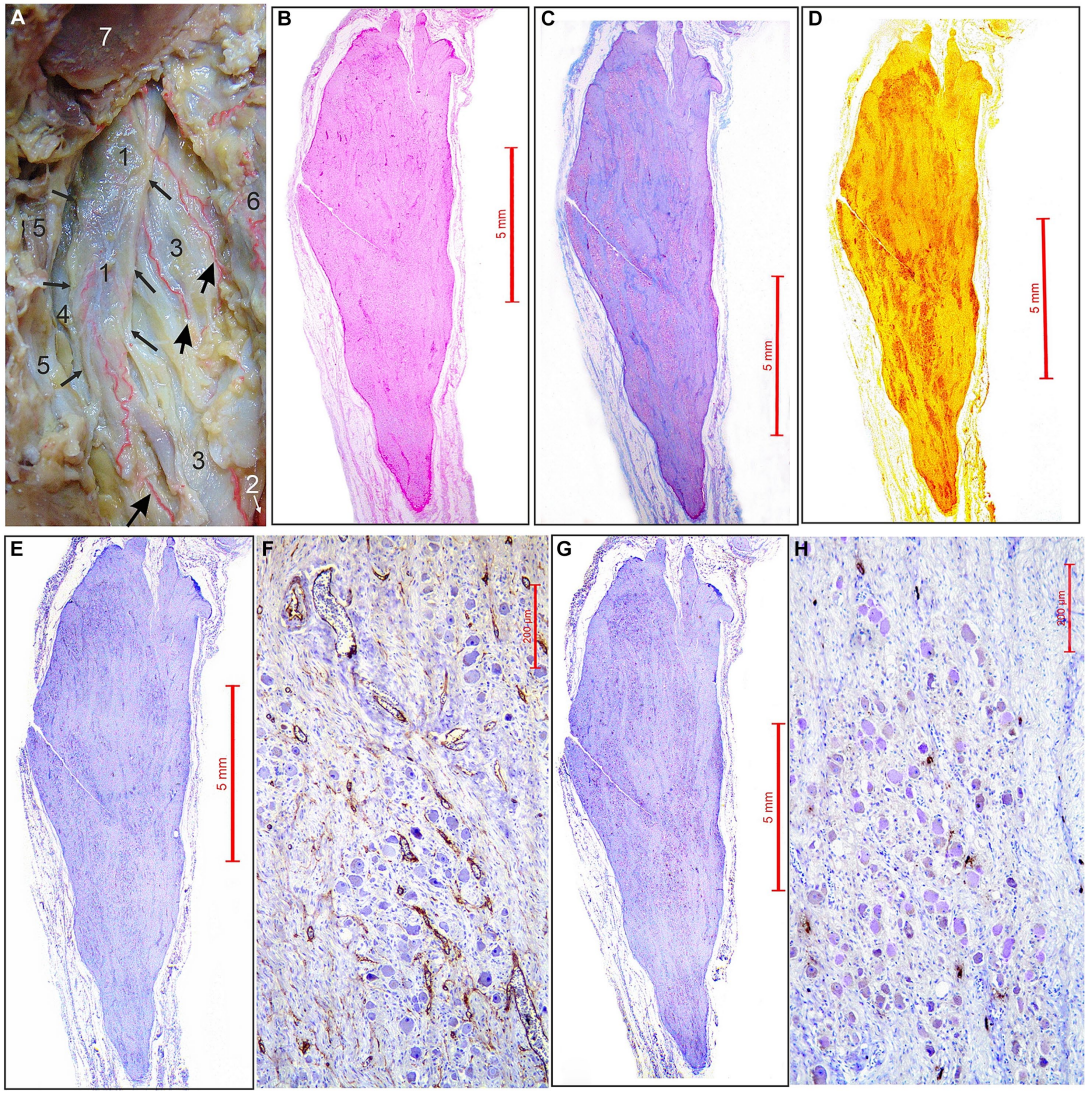
Arterial supply and micromorphological characteristics of the superior cervical sympathetic ganglion (SCSG). **(A)** SCSG (1) with borders indicated by small arrows receives three branches (large arrows) coming from the ascending pharyngeal artery (2), posterior to the internal carotid artery (3), vagus nerve (4) and internal jugular vein (5); posterior pharyngeal wall (6); occipital condyle, transected (7) (view from the back after disarticulation of the head and reflection of the pharyngeal wall anteriorly from the vertebral column). **(B)** SCSG specimen stained with hematoxilin and eosin (H&E) stain. **(C)** SCSG specimen stained with the Masson trichrome method. **(D)** SCSG specimen stained with silver staining method. **(E)** SCSG specimen and **(F)** small-magnification view showing vascular supply of the ganglionic tissue (CD34 immunostaining). **(G)** SCSG specimen and **(H)** small-magnification view showing mast cells related to the ganglionic tissue (mast cell tryptase immunostaining).

**Figure 2:**
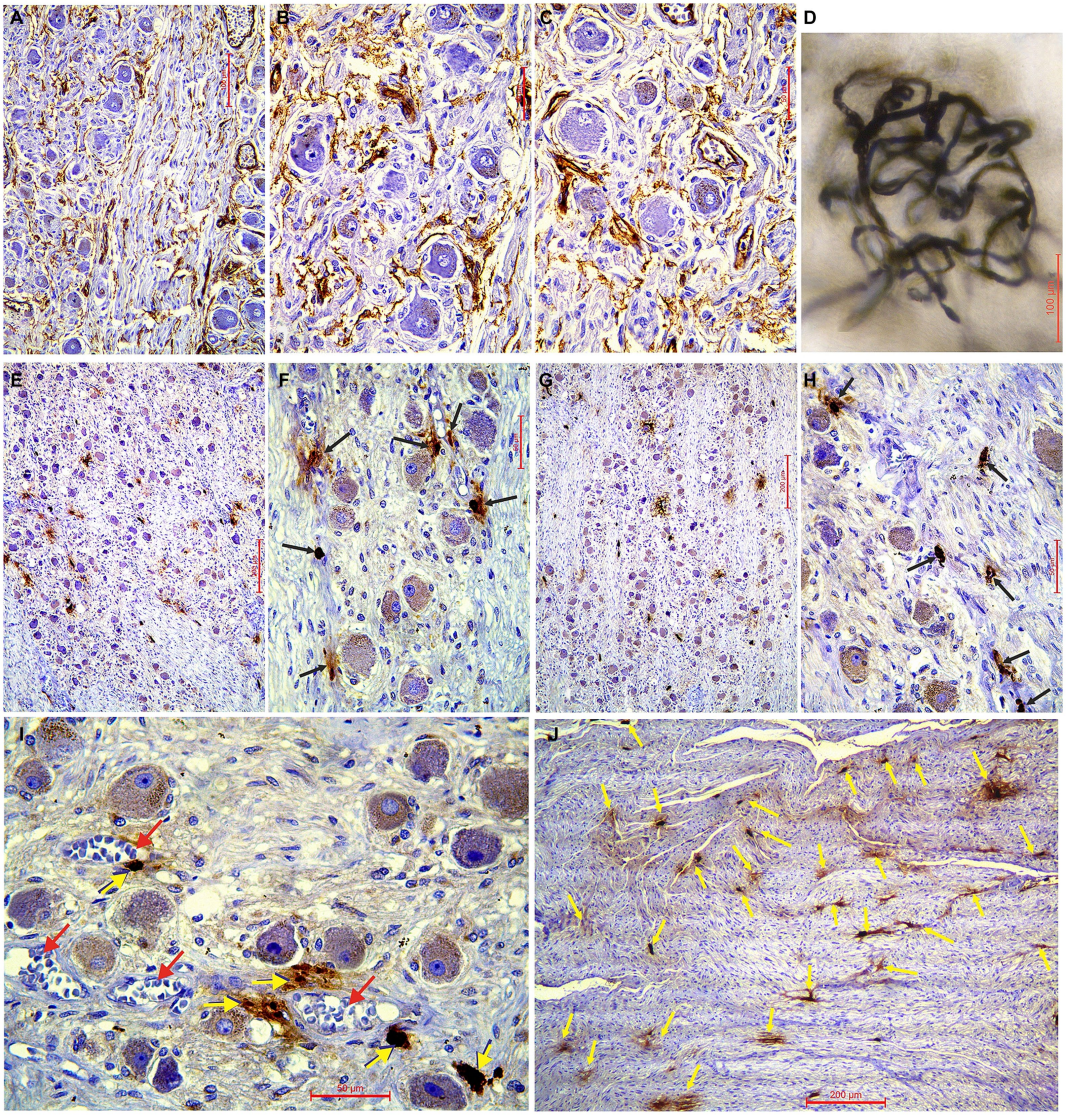
**(A–C)** Immunohistochemical characteristics of the superior cervical sympathetic ganglion (SCSG) blood supply (CD34 immunostaining), and **(E–H)** mast cells related to the human SCSG neurons (mast cell tryptase immunostaining). **(A)** Slender network of intraganglionic microvessels surrounding the ganglionic neurons of SCSG and parallel microvessels within the fascicle of axons and fibrous tissue. **(B)** Delicate plexiform microvessels of the upper part of SCSG fill the interneuronal spaces. **(C)** Dense network of microvessels of the lower part of SCSG covering the satellite glial cells and the deeper neurons. **(D)** Detail of higher magnification of the ganglionic capillary plexus injected with India ink and cleared in methyl salicylate. **(E)** Low-magnification of the upper part of SCSG specimen and **(F)** high-magnification view showing mast cells (arrows) related to the neurons (mast cell tryptase immunostaining). **(G)** Low-magnification of the lower part of SCSG specimen and **(H)** high-magnification view showing mast cells (arrows) related to the ganglionic tissue (mast cell tryptase immunostaining). **(I)** Tryptase-positive mast cells (yellow arrows) always in close vicinity to blood vessels (red arrows). **(J)** Numerous mast cells (yellow arrows) found between the axons of the internal carotid nerve at the exit from SCSG.

The ascending pharyngeal artery (APhA) in all five cases coursed superiorly, behind and laterally to the pharyngeal wall. Among the other branches APhA sent 3–4 branches for the supply of superior cervical sympathetic ganglion (SCSG) (Figure 1A). The ganglionic branches approached the anterior and posterior surfaces of the SCSG, and from the external capsular fibrous layer sent penetrating vessels, the arterioles into the stroma composed of clusters of neurons separated with the bands of axons and the fibrous tissue (Figures 1A,E,F, 2A–D). Finally, the intraganglionic small arteries, mostly capillaries, surrounded the clusters of ganglionic neurons forming a capillary loops around each neuron (Figure 2D).

### Density of microvessels in the SCSG

We used longitudinal sections of the whole human SCSGs specimens for the studies of their micromorphological characteristics (Figure 1). Blood vessels of the SCSG showed a strong immune reaction against CD34 protein in endothelial cells (Figures 1E,F, 2A–C). Each microscopic field of the SCSGs upper parts contained 67 to 104 microvessels (mean 83 ± 11.97). The mean number of microvessels presented in microscopic fields of the lower parts was 82.7 ± 10.2 (68–98). We found no statistically significant differences in MVD between the two parts of the SCSG, upper and lower (*p* > 0.05; *p* = 0.953) (Table 1) (Figures 2B,C). The distribution of microvessels in two parts of the SCSGs was uniform with no specific micromorphological differences (Figures 2D–F).

**Table 1:** Microvessel density (MVD), mast cell density (MCD), number of neurons, and distribution of three groups of neurons in two parts of the superior cervical sympathetic ganglions (SCSGs): upper and lower.

SCSGs	Upper parts	Lower parts
N^o^ of microvessels/field: min–max (M ± SD)	67–104 (83 ± 11.97)	68–98 (82.7 ± 10.2)
N^o^ of mast cells/field: min–max (M ± SD)	2–6 (4.5 ± 1.43)	3–6 (4.7 ± 0.95)
N^o^ of neurons/field: min–max (M ± SD)	13–24 (19.5 ± 3.14)	12–24 (19.8 ± 3.65)

Cell body diameter (μm)	Small (15–30)	Medium (30.1–40)	Large (40.1–55)	Small (15–30)	Medium (30.1–40)	Large (40.1–55)
N^o^ of neurons/field (M)	166	28	1	162	36	0
%	85.1%	14.3%	0.6%	81.8%	18.2%	0%
Total	195 (100%)			198 (100%)		

### Density of mast cells in the SCSG

We analyzed the distribution of mast cells in the SCSGs and periganglionic tissue (Figures 1G,H). The tryptase-positive brown mast cells (MCs), ovoid in shape, varied in mean diameter (Figures 2E,G). As shown in Table 2 each microscopic field of the upper parts of the SCSGs comprised an average of 4.5 ± 1.43 MC (from 2 to 6). The mast cell density (MCD) of the lower parts of the SCSGs was 4.7 ± 0.95 MC (from 3 to 6). We found no statistically significant differences between the MCD groups in two parts of the SCSGs (*p* > 0.05; *p* = 0.717) (Table 1) (Figures 2F,H).

**Table 2:** Metric characteristics of neurons in two parts of the SCSGs: upper and lower.

NEURONS Upper parts	M (μm)	SD (±μm)	SE (±μm)	Min (μm)	Max (μm)
R2	24.37	5.73	0.41	12.23	43.76
R1	25.7	8.08	0.58	12.86	72.24
RM	25.04	5.98	0.43	14.79	52.32
C	79.29	19.67	1.41	47.13	175.88
	**M (μm**^**2**^**)**	**SD (** ± **μm**^**2**^**)**	**SE (** ± **μm**^**2**^**)**	**Min (μm**^**2**^**)**	**Max (μm**^**2**^**)**
Area	509.5	246.2	17.63	166.542	1838.886

NEURONS Lower parts	M (μm)	SD (±μm)	SE (±μm)	Min (μm)	Max (μm)
R2	24.24	6.25	0.44	11.76	48.97
R1	26.33	5.35	0.38	12.15	39.82
RM	25.58	5.10	0.36	13.71	39.16
C	79.36	16.15	1.15	43.08	124.68
	**M (μm**^**2**^**)**	**SD (** ± **μm**^**2**^**)**	**SE (** ± **μm**^**2**^**)**	**Min (μm**^**2**^**)**	**Max (μm**^**2**^**)**
Area	527.62	208.64	14.83	147.484	1172.155

R2-shorter diameter; R1-longer diameter; RM-mean diameter; C-circumference; M-mean, SD-standard deviation; SE-standard error.

Tryptase-positive mast cells (MCs) were very frequently found between the axons of outgoing sympathetic nerves, always adjacent microvessels (Figures 2I,J), and in the periganglionic connective tissue.

### Density of neurons in the SCSG

The number of ganglionic neurons in the same microscopic fields of the mentioned slices ranged from 13–24 (mean 19.5 ± 3.14) for the upper parts of the SCSGs, and from 12–24 (mean 19.8 ± 3.65) for the lower parts of the SCSGs (Table 1) (Figures 2D–F). Again, we showed that the distribution of the neurons was not significantly different in the two parts of the SCSGs (*p* > 0.05; *p* = 0.846).

**Figure 3:**
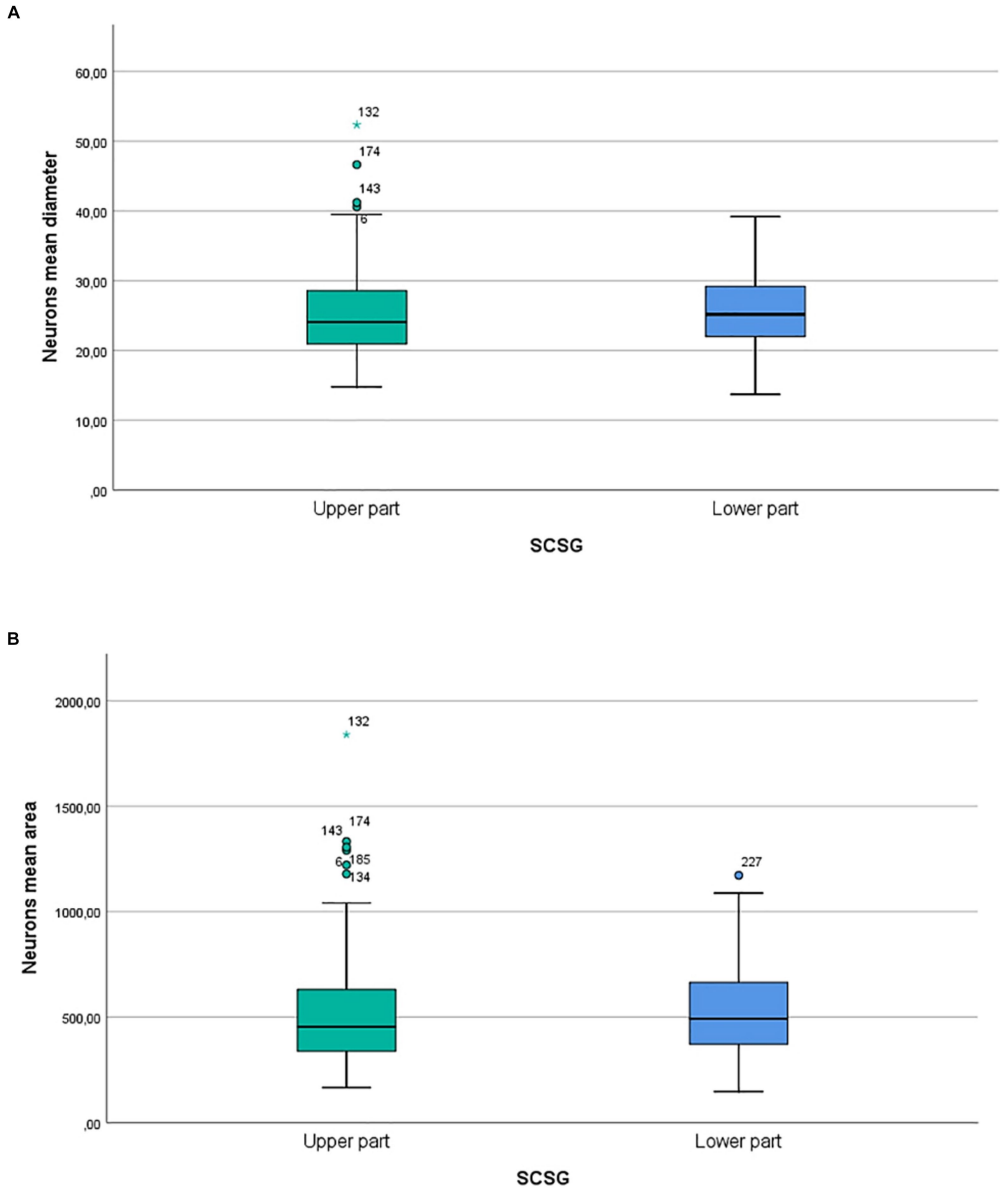
**(A)** Comparison of neurons mean diameters (μm) in two parts of SCSG: upper and lower. No significant differences were detected between two groups (*p* > 0.05; *p* = 0.333). **(B)** Comparison of neurons mean cross-sectional areas (μm^2^) in two parts of SCSG: upper and lower. No significant differences were detected between two groups (*p* > 0.05; *p* = 0.431).

Our micromorphometric analysis of the ganglionic neurons of SCSGs included the measurements of the longer and shorter diameters of neurons and the calculation of mean diameter of nerve cells. The ganglionic neurons varied in mean diameter from 25.04 μm for the upper parts to 25.58 μm for the lower parts of SCSGs. Comparing the mean diameter of 195 ganglionic neurons of the upper parts of SCSGs with the mean diameter of 198 ganglionic neurons of the lower parts of SCSGs we found no significant statistical difference in two ganglionic parts (*p* > 0.05; *p* = 0.333) (Table 2; Figure 3).

We formed three main groups of SCSGs neurons: small (15–30 μm), medium-sized (30.1–40 μm), and large neurons (40.1–55 μm). According to this classification small-sized neurons were the largest group of cells within human SCSGs, 85.1% in the upper parts, and 81.8% of neurons in the lower parts of ganglions. Medium-sized neurons were present in a lower number, 14.3% in the upper parts, and 18.2% of neurons in the lower parts of ganglions (Table 1; Figure 2).

The ganglionic neurons varied in mean cross-sectional area from 509.5 μm^2^ for the upper parts to 527.62 μm^2^ for the lower parts of SCSGs. Finally, comparing the mean cross-sectional areas of 195 neurons of the upper parts with the 198 neurons of the lower parts of SCSGs we also found no significant statistical difference in two analyzed parts (*p* > 0.05; *p* = 0.431) (Table 2; Figure 3).

## Discussion

Anatomic studies describing the ascending pharyngeal artery (APhA) are very rare, and the descriptions specific to the SCSG supply are incomplete or missing. The researches agreed that, among the other branches, the APhA send twigs for the vascularization of the human SCSG (Tubbs et al., 2002; Cavalcanti et al., 2009). Our results indicate that the SCSG received 3–4 arterial branches from the APhA which approached the ganglionic tissue from the anterior and posterior sides, curving over the ganlion, sending pentrating arterioles throughout the ganglionic stroma. Microvasculature of the SCSG attracted our attention, especially the intraganglionic small arteries, mostly capillaries, surrounded clusters of ganglionic neurons, forming a network, a capillary loops around each of the ganglionic neuron. Sections of one SCSG, of specimen injected with India ink and melted gelatin were cleared by a Spalteholz technique and we analyzed tortuous microvessels coils in the form of nests over spherical SGCs-ganglionic cells units. The intraganglionic capillary plexus exposed and analyzed under the transmitted light visually appeared uniform, there was a homogenous vascular pattern within the SCSG. A group of authors also stated that the intraganglionar microcirculation, the microcirculatory bed, is composed of capillaries that form spatial loops surrounding the trigeminal ganglion somata (Smoliar et al., 1998). Comparable results were obtained in our previous study that followed the similar protocol of research and the subject was the trigeminal ganglion (TG). According to this study, a very dense and curved intraganglionic capillary network was visually apparent with a uniform density of microvessels throughout the trigeminal ganglion (Ćetković et al., 2020).

Our immunohistochemical examination of the SCSGs specimens exposed rich microvasculature coils, mainly capillaries and precapillaries with diameters from 5.5 μm to 8.5 μm, around the ganglionic cells. The microvessels were identified by immunostained whole transverse sections, or variously transected independent vessels, or parts of their vascular walls. The mean number of CD34-positive microvessels varied per microscopic field: 83 for the upper parts, and 82.7 for the lower part of the SCSGs. The density of CD34-positive microvessels was not significantly different (*p* = 0.953) comparing two observed parts of the SCSGs. The uniform distribution of microvessels throughout the SCSG confirmed the existence of a functionally homogenous vascular pattern for two parts of the ganglion: upper and lower. Comparing the mean number of CD34-positive microvessels of the whole SCSGs, 82.85, with the mean number of CD34-positive microvessels of the whole TGs, 102.86, found in our previous study (Mirčić et al., 2021), we confirmed the higher MVD in the TGs, probably because of the higher oxygen and metabolic demands of sensory ganglionic cells then the sympathetic ganglionic cells.

This study indicated the homogenous presence of tryptase-positive mast cells in upper and lower parts of the SCSGs, the MCD had a value of 4.5 and 4.7, respectively. We can compare the higher average value of MCD in the SCSGs with the lower presence of mast cells found in the TG in our previous study with an average MCD of 1.35 (Mirčić et al., 2021). The present results suggested probably that in response to psychological stress with the release of stress hormones, the activation of mast cells is important for the stimulation of the sympathetic ganglionic neurons of the SCSG (Van der Kleij and Bienenstock, 2005). We agree that “structure determines function,” the arrangement and number of mast cells have a specific influence on the activity of ganglionic neurons in the SCSG. Increased number of MCs related to the axons and neurons, possibly modulate function of nerve cells, and have a role in nociception and hyperalgesia (Koyuncu Irmak et al., 2019). This study suggests that MCs are important for the further understanding of the patophysiology of headaches and pain conditions, together with blood vessels and sensory nerves. Inhibition of MCs degranulation should be possible therapeutic treatment for these conditions.

The ganglionic cells of the cat and rat were traditionally classified in three distinct groups of TG neurons: small (20–30 μm), medium-sized (30–50 μm), and large neurons (50–80 μm) (Lazarov, 2002). Our similar classification, and micromorphometric analyses showed that small-sized neurons were the dominant group of cells within human SCSGs. In the upper parts of the SCSGs 85.1% of neurons were small, and in the lower parts of ganglions 81.8% of neurons, the rest of cells belonged to the medium-sized neurons. Comparing the mean number, the mean diameter, and the mean cross-sectional areas of 195 ganglionic neurons of the upper parts of SCSGs with the values of 198 ganglionic neurons of the lower parts of SCSGs we found no significant statistical differences in two ganglionic parts.

The human SCSGs neurons, of different size and number, contain different neuropeptides: neuropeptide Y (NPY), vasoactive intestinal polypeptide (VIP), calcitonin gene-related peptide (CGRP), somatostatin (SOM), etc., (Baffi et al., 1992; Miyauchi et al., 2001). Analysis and comparison of NPY immunoreactivity of SCSG neurons in the rat indicated that NPY-positive neurons were smaller than NPY-negative neurons at more distal position in the SCSG, close to the external carotid nerve (Headley et al., 2005). The goal of our future study should be to identify, quantify and compare the immunoreactive neurons in the proximal and distal parts of human SCSG.

In conclusion the density of CD34-positive microvessels, the density of tryptase-positive mast cells, and the mean number of ganglionic neurons and their metric characteristics, showed no significant differences in the upper and lower parts of the SCSG. Our results proved that the distribution of microvessels, mast cells, and neurons in upper and lower parts of the SCSG was homogeneous with no specific micromorphological differences in distribution. More than 80% of neurons belonged to the small size cells in both parts of the SCSG.

## Data availability statement

The raw data supporting the conclusions of this article will be made available by the authors, without undue reservation.

## Author contributions

JB: Conceptualization, Investigation, Software, Writing – original draft. MM: Conceptualization, Data curation, Investigation, Methodology, Project administration, Validation, Writing – review & editing. ML: Formal analysis, Supervision, Validation, Writing – review & editing. LP: Formal analysis, Supervision, Validation, Writing – review & editing. NiB: Data curation, Project administration, Writing – review & editing. MV: Data curation, Project administration, Writing – review & editing. DA: Data curation, Formal analysis, Resources, Writing – review & editing. DĆ: Data curation, Project administration, Visualization, Writing – review & editing. NeB: Data curation, Formal analysis, Methodology, Writing – review & editing. AD: Conceptualization, Methodology, Validation, Writing – original draft, Conceptualization, Methodology, Validation, Writing – original draft. MĆ: Conceptualization, Formal analysis, Methodology, Writing – original draft, Writing – review & editing.
